# The Association of Income with Health Behavior Change and Disease Monitoring among Patients with Chronic Disease

**DOI:** 10.1371/journal.pone.0094007

**Published:** 2014-04-10

**Authors:** David JT. Campbell, Paul E. Ronksley, Braden J. Manns, Marcello Tonelli, Claudia Sanmartin, Robert G. Weaver, Deirdre Hennessy, Kathryn King-Shier, Tavis Campbell, Brenda R. Hemmelgarn, for the Interdisciplinary Chronic Disease Collaboration

**Affiliations:** 1 Department of Community Health Sciences, University of Calgary, Calgary, Alberta, Canada; 2 Department of Medicine, University of Calgary, Calgary, Alberta, Canada; 3 Ottawa Hospital Research Institute, Ottawa, Ontario, Canada; 4 Statistics Canada, Health Analysis Division, Ottawa, Ontario, Canada; 5 Institute of Public Health, University of Calgary, Calgary, Alberta, Canada; 6 Libin Cardiovascular Institute, University of Calgary, Calgary, Alberta, Canada; 7 Department of Medicine, University of Alberta, Edmonton, Alberta, Canada; 8 Faculty of Nursing, University of Calgary, Calgary, Alberta, Canada; 9 Department of Psychology, University of Calgary, Calgary, Alberta, Canada; Oregon Health & Science University, United States of America

## Abstract

**Background:**

Management of chronic diseases requires patients to adhere to recommended health behavior change and complete tests for monitoring. While studies have shown an association between low income and lack of adherence, the reasons why people with low income may be less likely to adhere are unclear. We sought to determine the association between household income and receipt of health behavior change advice, adherence to advice, receipt of recommended monitoring tests, and self-reported reasons for non-adherence/non-receipt.

**Methods:**

We conducted a population-weighted survey, with 1849 respondents with cardiovascular-related chronic diseases (heart disease, hypertension, diabetes, stroke) from Western Canada (n = 1849). We used log-binomial regression to examine the association between household income and the outcome variables of interest: receipt of advice for and adherence to health behavior change (sodium reduction, dietary improvement, increased physical activity, smoking cessation, weight loss), reasons for non-adherence, receipt of recommended monitoring tests (cholesterol, blood glucose, blood pressure), and reasons for non-receipt of tests.

**Results:**

Behavior change advice was received equally by both low and high income respondents. Low income respondents were more likely than those with high income to not adhere to recommendations regarding smoking cessation (adjusted prevalence rate ratio (PRR): 1.55, 95%CI: 1.09–2.20), and more likely to not receive measurements of blood cholesterol (PRR: 1.72, 95%CI 1.24–2.40) or glucose (PRR: 1.80, 95%CI: 1.26–2.58). Those with low income were less likely to state that non-adherence/non-receipt was due to personal choice, and more likely to state that it was due to an extrinsic factor, such as cost or lack of accessibility.

**Conclusions:**

There are important income-related differences in the patterns of health behavior change and disease monitoring, as well as reasons for non-adherence or non-receipt. Among those with low income, adherence to health behavior change and monitoring may be improved by addressing modifiable barriers such as cost and access.

## Introduction

Optimal management of chronic disease is complex, requiring involvement from practitioners and patients. Models of care to guide chronic disease management, including the Chronic Care Model [1], emphasize the need for close interactions between ‘a prepared, proactive practice team’ and ‘informed, activated patients’. The responsibilities of these parties vary, with healthcare providers responsible for prescribing evidence-based therapies, recommending disease monitoring, and counseling patients on healthy lifestyle choices, while patients are expected to adhere to prescribed therapy including making health behavior changes, and complete recommended tests for disease monitoring.

Among those with cardiovascular-related chronic diseases (such as diabetes, hypertension, heart disease and stroke), health behavior change, including sodium reduction [2]–[4], dietary improvement [5], exercise [6]–[7], smoking cessation [8] and weight loss [7], [9], has been associated with a reduced risk of cardiovascular events and mortality. Similarly, in high-risk populations, cholesterol testing [10]–[11], blood glucose testing [12] and blood pressure measurement [13] are associated with improved outcomes. Unfortunately, many patients do not make health behavior changes or receive the recommended monitoring. This can be due to provider-related factors (e.g. lack of communication skills [14] and failure to advise patients of the necessary health behavior changes or tests for disease monitoring) or patient-related factors (e.g. low level of education or health literacy [15], incongruent health beliefs [16], or simply choosing to not adhere to recommendations).

Population health experts suggest that an individual’s ability to adhere to such recommendations is also constrained by external factors, referred to as the social determinants of health – including socioeconomic status (SES) [17]–[18]. Although SES is a construct which involves several of the social determinants of health including income, ethnicity, immigration status, education level, and social class [19], SES is often closely tied to income. Income is considered to be among the most important of the social determinants [20]–[21], and often used as a proxy for SES [22]–[24]. A 2010 systematic review identified differences in the uptake of behavior changes by SES. However, the authors noted a paucity of studies examining the relationship between income and adherence to these strategies [25]. While several authors have explored the association between SES and cancer screening [26]–[28], the only study of which we are aware examining the relationship between SES (or income) and cardiovascular preventive care was a clinic-based study from a single geographic region with limited generalizability [29]. Furthermore, patient-described reasons for non-adherence/non-receipt and their association with income in particular have received little attention in the literature.

We were interested in examining the relationship between household income and adherence to health behavior change and completion of monitoring tests among patients with chronic disease. Our objectives were to: (1) determine the proportion of patients with chronic disease who received physician-directed advice for health behavior change, whether they were adherent to these recommendations, and reasons for non-adherence; (2) determine the proportion of patients who received recommended disease monitoring and reasons for those who did not have testing done; and (3) determine if low income status was associated with non-adherence to health behavior change or non-completion of monitoring tests.

## Methods

### Study Population

From February 1 to March 31 2012, Statistics Canada administered a special survey designed by the Interdisciplinary Chronic Disease Collaboration (www.ICDC.ca)[30], using computer assisted telephone interviews. The sampling frame consisted of all 2011 Canadian Community Health Survey (CCHS)[31] respondents who resided in British Columbia, Alberta, Saskatchewan or Manitoba, were over age 40 and self-reported having at least one of: heart disease, stroke, diabetes, or hypertension.

### Survey Details

We designed our survey to include questions about processes of care and potential barriers to care for people with at least one of the chronic diseases of interest. In accordance with Statistics Canada procedures, the survey underwent pilot testing and revisions to ensure the questionnaire content was relevant to the population [32].

This project was approved by the Conjoint Health Research Ethics Board of the University of Calgary and the Health Research Ethics Board of the University of Alberta and the authors followed all procedures and stipulations of these boards. Informed consent was obtained verbally from all survey participants. Verbal consent was used rather than written consent as the survey was administered via telephone. The consent dialogue was recorded for record keeping purposes. Participants also agreed to have their responses linked to responses from the 2011 Canadian Community Health Survey. These procedures were approved by both institutions' ethics boards.

### Variables of Interest

#### Exposure: income status

Respondents to the CCHS were asked about their household income. The majority of respondents (70%) provided their annual household income and for the remainder, income was imputed using a nearest neighbor technique including postal code median income, household size and approximate household income range [33]. We defined low income as a household income of <$30,000 CAD, which approximates the low-income cut-offs for a family of 2–3 people, as defined by Statistics Canada [34].

#### Sodium restriction, dietary modification, and physical activity

In separate questions, respondents were asked if their doctor or other health professional had ever advised them to reduce salt intake, consume a healthier diet or participate in physical activities. All respondents were then asked if they ever made these modifications. Those who indicated yes were asked if they maintained the modification ‘all of the time’, ‘most of the time’, ‘some of the time’, or ‘none of the time’. We defined non-adherence to making a behavior change as responding ‘no’ to ever having made a change, or as less than ‘all of the time’ to the maintenance question [35].

#### Smoking cessation

Respondents who indicated that they had smoked tobacco since their chronic disease diagnosis were asked if they had ever received advice to quit or reduce smoking. The ensuing questions were identical to those described above for the other health behavior changes.

#### Weight loss

All respondents were asked if they had received advice to lose weight to help manage their chronic condition. The ensuing questions were identical to those described above for the other health behaviors. We excluded all those whose body mass index (BMI) fell in the normal or underweight categories (<25.0 kg/m^2^).

#### Reasons for non-adherence

Those who reported they did not adhere to a behavior modification were subsequently asked to provide reasons for failing to adhere. These responses were categorized in a manner consistent with prior work in the area [36]–[37]: personal choice, not available/accessible, financial/cost, inadequate patient knowledge, and other (Appendix S1). ‘Personal choice’ was the only category cited frequently enough to be used consistently in the analysis of income and each of the outcome measures, although we also considered the most common answer other than ‘personal choice’ for each outcome measure.

#### Receipt of monitoring tests

We also assessed receipt of recommended monitoring tests among respondents at high cardiovascular risk. The US-based National Cholesterol Education Program recommends that patients who are using lipid lowering therapy have their cholesterol levels checked at least annually [38]. The American Diabetes Association recommends that all adults over the age of 45 undergo blood glucose screening tests every three years, or more frequently for those at high risk [39]. According to the National High Blood Pressure Education Program, high risk individuals should have at least annual blood pressure measurement [40].

We defined “high cardiovascular risk” using an algorithm modified from the Canadian Diabetes Association practice guidelines [41] and Canadian Hypertension Education Program recommendations [42], acknowledging that we lacked some clinical information required for this determination (e.g. blood pressure, family history, symptoms). This high risk group of persons with at least one chronic disease included: those who self-reported having heart disease or stroke; those who had both hypertension and diabetes; current smokers; those with hypertension only who were greater than 55 years old; and those with diabetes only who were greater than 45 years old for males or 50 years old for females.

In separate questions, respondents classified as high risk were asked if they had their cholesterol, blood glucose and blood pressure checked at least once within the 12 previous months. Those who stated that they had not had each of these were classified as not receiving that test. A separate variable was created identifying those who had received all three monitoring tests.

#### Reasons for non-completion of monitoring

Those who indicated they had not received each monitoring test were subsequently asked the reason, with the responses categorized as described above (Appendix S1).

#### Covariates

Potential covariates, as determined from existing literature regarding predictors of non-adherence to behavior changes [43]–[45], included age (≥ and <65 years), sex, obesity (BMI >30, corrected for self-report [46]), having more than one of the four chronic conditions of interest (hereafter called multimorbidity), having a regular medical doctor, and other socio-demographic characteristics (e.g. education level, immigration status). Socio-demographic characteristics were categorized as presented in Table 1. Definitions of each covariate of interest are described elsewhere [31].

**Table 1:** Baseline Characteristics, overall and stratified by income status.

Variable		Total	Income ≥$30,000	Income <$30,000.	Unadjusted
		(n = 1849)	78.2%	21.8%	
		% (95% CI)^†^	% (95%CI)	% (95%CI)	PRR (95% CI)
Age (yrs)	<65	48.8 (45.7–52.1)	55.1 (51.3–59.0)	26.4 (19.6–33.1)	
	65+	52.2 (47.9–54.3)	44.9 (41.0–48.7)	73.6 (66.9–80.4)	**1.64 (1.43–1.88)**
Sex	Female	50.1 (46.2–54.0)	46.5 (42.0–51.1)	62.8 (55.4–70.3)	
	Male	49.9 (46.0–53.8)	53.5 (48.9–58.0)	37.2 (29.7–44.6)	**0.70 (0.56–0.87)**
Region	Urban	82.5 (79.5–85.4)	82.0 (78.4–85.5)	84.3 (80.0–88.7)	
	Rural	17.5 (14.6–20.5)	18.0 (14.5–21.6)	15.7 (11.3–20.0)	0.87 (0.62–1.22)
Number of Chronic conditions	1 Chronic condition	67.8 (64.8–70.8)	70.6 (67.1–74.1)	57.7 (50.7–64.7)	
	2+ Chronic conditions	32.2 (29.2–35.2)	23.4 (25.9–32.9)	42.3 (35.3–49.3)	**1.44 (1.17–1.78)**
Marital Status	Widowed/Sep/Div/Single	33.1 (29.4–36.8)	25.1 (21.2–29.1)	61.5 (53.4–69.7)	
	Married/Common-law	66.9 (63.2–70.6)	74.9 (70.9–78.8)	38.5 (30.3–46.6)	**0.51 (0.41–0.64)**
Level of Education	Less than High school	21.3 (18.6–24.1)	16.7 (13.7–19.7)	38.1 (31.7–44.5)	
	At least High school graduate	78.7 (75.9–81.4)	83.3(80.3–86.3)	61.9 (55.5–68.3)	**0.74 (0.67–0.83)**
Corrected BMI [46]	<30 kg/m2	59.9 (56.0–53.8)	59.8 (55.4–64.3)	60.3 (52.6–68.1)	
	≥30 kg/m2	40.1 (36.2–44.0)	40.2 (35.7–44.6)	39.7 (31.9–47.4)	0.99 (0.79–1.24)
Immigration Status	Born outside of Canada	23.8 (20.3–27.7)	22.2 (17.9–26.4)	30.1 (21.4–38.9)	
	Born in Canada	76.2 (72.3–79.7)	77.8 (73.6–82.1)	69.9 (61.1–78.6)	0.90 (0.78–1.03)
Province	Alberta	31.7 (28.8–34.6)	32.9 (29.2–36.6)	27.3 (20.6–34.0)	
	Other (SK, BC, MB)	68.3 (65.4–71.2)	67.1 (63.4–70.8)	72.7 (66.0–79.4)	1.08 (0.96–1.22)
Ethnicity	White	87.0 (83.4–89.9)	87.0 (83.2–90.8)	85.6 (80.1–91.0)	
	Aboriginal/Other	13.0 (10.1–16.6)	13.0 (9.2–16.8)	14.4 (9.0–19.9)	1.11 (0.68–1.81)
Has Regular Medical Doctor	No	4.9 (2.9–6.8)	4.8 (2.5–7.1)*	5.0 (1.9–8.1)*	
	Yes	95.1 (93.2–97.1)	95.2 (92.9–97.5)	95.0 (91.9–98.1)	1.00 (0.96–1.04)

*CV = 16.5–33 (Interpret with caution); Abbreviations: CI – confidence interval; PRR – prevalence rate ratio; sep/div – separated or divorced; SK – Saskatchewan; MB – Manitoba; BC – British Columbia.

### Statistical Analyses

We used proportions to describe each outcome: receipt of behavior change advice, adherence to health behavior change, stating personal choice reasons for non-adherence, receipt of monitoring tests, and stating personal choice as reasons for non-receipt, and stratified by household income (dichotomized at <$30,000, and ≥$30,000). Frequency weights were calculated by Statistics Canada to account for non-representative sampling and to reflect the adult population with chronic disease in the four Western provinces [47]. Bootstrapping procedures with 500 replications were used to calculate standard errors and confidence intervals around the estimates. As recommended by Statistics Canada, the coefficient of variation (CV) was used to determine the reliability of reported proportions [48].

We used log-binomial regression models to calculate adjusted prevalence rate ratios (PRR) to determine the association between low income status and each outcome. Initial models included each of the covariates that significantly differed across income categories (age, BMI, multimorbidity, education, sex), as well as interaction terms between age and income, and between sex and income. Backwards elimination techniques were used to obtain the most parsimonious model, with interaction terms eliminated based on non-significant Wald tests (p>0.05). Confounding was assessed using backward elimination and a change in the estimate for the exposure (>10%). We noted no effect modification by age or gender. Age was the only significant confounding variable; therefore the final models presented are age-adjusted. All analyses were performed using STATA 11.0 (Statacorp, College Station, TX).

## Results

### Baseline Characteristics

From our sampling frame of 2316 individuals, we obtained 1849 completed surveys, yielding a response rate of 80%. The majority of respondents had only one chronic condition of interest (68%), were married/common-law (67%), lived in urban areas (83%), and were above the low income threshold (78%). There were several significant differences between income groups (Table 1), with the low income group more likely to be older, female, single/widowed/separated, and have a lower level of education.

### Receipt of Health Behavior Change Advice

Rates of receiving health behavior change advice were relatively high (between 61–88%), with smoking cessation being the most common advice provided (88%) (Table 2). After adjusting for age, there were no significant differences in the likelihood of receiving behavior change advice by income category.

**Table 2:** Proportion of population who received recommendations for health behavior change, overall and by income status.

	Overall	≥$30,000	<$30,000	Age adjusted
	% (95%CI)	% (95%CI)	% (95%CI)	PRR (95%CI)
**Sodium Reduction**	61.0 (57.2–64.7)	61.2 (57.0–65.4)	60.1 (53.0–67.1)	1.02 (0.89–1.16)
**Diet Modification**	63.5 (59.7–67.3)	65.1 (60.7–70.0)	57.6 (50.0–65.2)	0.92 (0.79–1.07)
**Increased Physical Activity**	74.8 (71.3–78.3)	76.1 (72.2–80.1)	70.0 (63.0–77.0)	0.97 (0.87–1.07)
**Smoking Cessation among smokers (26.1%)**	88.4 (84.5–92.2)	88.6 (83.9–93.3)	87.6 (80.6–94.6)	1.00 (0.91–1.10)
**Weight Loss among overweight and obese individuals (76.8%)**	45.3 (41.4–49.2)	47.8 (43.1–52.5)	35.9 (29.0–42.8)	0.84 (0.67–1.06)

Abbreviations: CI – confidence interval; PRR – prevalence rate ratio.

### Non-adherence to Health Behavior Change Advice

**Figure 1:**
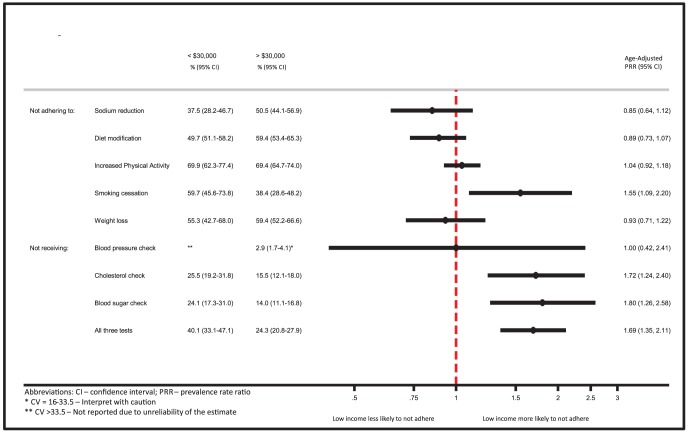
Non-adherence to lifestyle modifications or non-receipt of monitoring tests: Low income vs. High income.

Among those who received advice, there was a high rate of non-adherence (ranging from 48–70%) (Figure 1). After adjustment, those with low income were significantly more likely to not adhere to recommendations to reduce or quit smoking tobacco (adjusted PRR: 1.55; 95%CI: 1.09–2.20). There were no significant differences between income categories with regard to adherence to the other health behaviors.

### Non-receipt of Recommended Monitoring Tests

Among those at high cardiovascular risk, respondents with low income were more likely to not receive all three monitoring tests within the previous year, compared to those with higher income (adjusted PRR: 1.69, 95%CI: 1.35–2.11) (Figure 1). When considering each test individually, low income respondents were more likely to not have a cholesterol measurement (adjusted PRR: 1.72; 95%CI: 1.24–2.40) or blood glucose measurement (adjusted PRR: 1.80; 95%CI: 1.26–2.58). There was no difference in likelihood of having blood pressure measured, by income.

### Reasons for Non-adherence or Non-receipt

**Figure 2:**
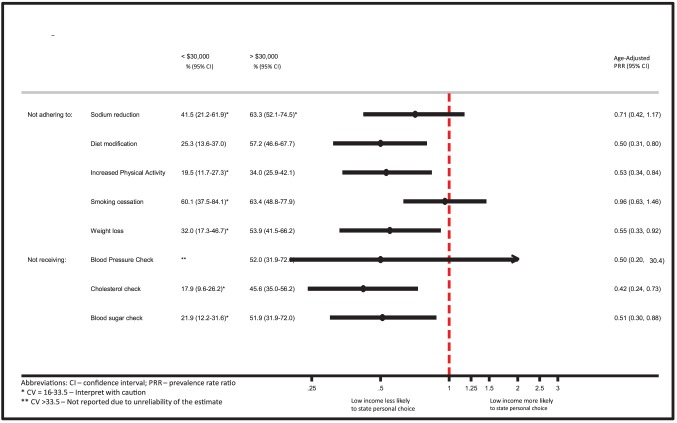
Noting “personal choice” as reason for non-adherence to health behavior change or non-receipt of monitoring tests: Low income vs. High income.

Compared to those with high income, respondents with low income were significantly less likely to report personal choice as a reason for not adhering to several health behavior changes, including: diet (adjusted PRR: 0.50; 95%CI: 0.31–0.80), exercise (adjusted PRR: 0.53; 95%CI: 0.34–0.84), and weight loss (adjusted PRR: 0.55; 95%CI: 0.33–0.92) (Figure 2). Similarly, those with low income were less likely to identify personal choice as the reason for failing to have their cholesterol (adjusted PRR: 0.42; 95%CI 0.24–0.73) and blood glucose measured (adjusted PRR: 0.51; 95%CI: 0.30–0.88) (Figure 2).

**Figure 3:**
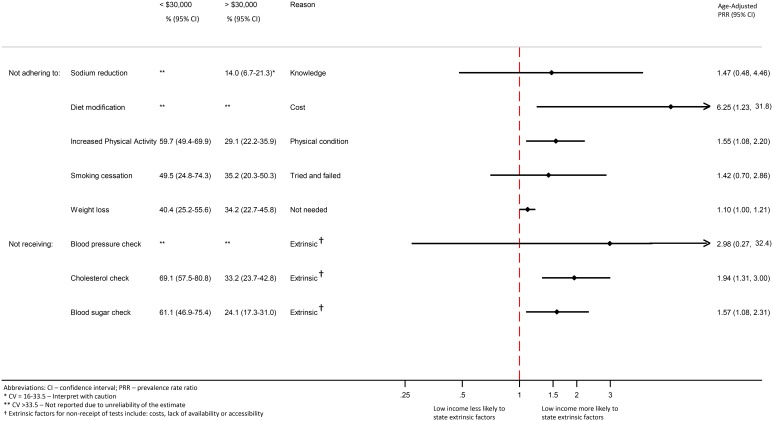
Noting non-personal choice reasons for non-adherence to health behavior change or non-receipt of monitoring tests: Low income vs. High income.

When considering other reasons for non-adherence or non-receipt of recommendations and monitoring, compared to those with high income, low income respondents were significantly more likely to report non-personal choice reasons for several domains(Figure 3): physical activity (physical condition, adjusted PRR: 1.55, 95%CI: 1.08–2.22), weight loss (lack of perceived need, adjusted PRR: 1.10, 95%CI: 1.00–1.21), cholesterol measurement (extrinsic reasons, adjusted PRR: 1.94, 95%CI: 1.31–3.00) and blood glucose measurement.

## Discussion

In our survey of adults with chronic disease we found that a large proportion of the population received advice regarding health behavior change. While the receipt of behavior change advice did not differ by income, there was variability in terms of adherence, and reasons for non-adherence, by income for some but not all of the variables of interest. Those with low income were less likely to adhere to smoking cessation and less likely to receive monitoring tests including blood glucose and cholesterol. The reason for non-adherence and non-receipt among the low income group was less likely to be related to personal choice, and was more likely to be related to extrinsic factors including cost, and availability of services across several of the outcome measures.

We found that rates of receiving advice for behavior change for this population with chronic disease were higher than those reported for the general population [49]–[50], although similar to what has been reported in other chronic disease populations [51]–[54]. The only difference in adherence to health behavior change by income category that we detected was for smoking, with the low income group less likely to stop smoking. This phenomenon may arise for several reasons, such as fear of judgment if they fail, and lack of knowledge associated with lower socioeconomic status [55]. Furthermore, those with low income often have weaker social support networks which if present may enable an individual to stop engaging in an addictive behavior such as smoking. A similar association between low income status and failure to quit smoking has been previously documented [56]–[59]. Also consistent with prior literature [29], [60]–[62], we found that those with low income were less likely to have monitoring tests, such as cholesterol and blood glucose checks, but not blood pressure monitoring. The reason for the differences in these monitoring tests may well have to do with the complexity of accomplishing them. Blood pressure checks can be quickly obtained in pharmacies, fire stations, physician offices or at home for those with blood pressure cuffs. In contrast, serum laboratory investigations (such as blood glucose or cholesterol), require multiple steps including an appointment with a family physician, a clinic visit, receipt of a laboratory requisition, attendance at a lab for the test to be drawn, and then follow-up with the ordering physician. The differences in time and resource intensity of these activities may be responsible for the differential association with income and serum monitoring tests compared with blood pressure monitoring.

Importantly our survey assessed reasons for non-adherence. We found that among the low income group non-adherence did not appear to be related to personal choice, but was reported to be related to extrinsic factors such as cost and availability of dietary modification and of monitoring tests. Given that the costs of such tests are covered by provincial health insurance, the reasons for the lack of monitoring may relate to indirect expenditures associated with completing these tests (e.g., travel costs, childcare, requiring time away from work). The fact that respondents with low income were less likely to attribute their inability to modify their lifestyle and receive monitoring tests to personal choice factors suggests that there are potentially modifiable barriers which preclude this group from fully participating in self-management and from receiving required disease monitoring. This is consistent with work produced by social scientists and population health researchers indicating that those with low income are predominantly disadvantaged by structural factors, rather than choosing to disregard health advice [63]. It is important to recognize that those with low income were more likely to state that external factors, such as cost, were responsible for their non-completion of monitoring tests, they were also less likely to adhere to smoking cessation. The cost savings of quitting tobacco use would far outweigh the expenses required to complete the monitoring tests. This dichotomy highlights the subjective nature of costs, and the fact that in health decision making the patient’s perception of cost may represent far more than simply absolute financial costs [64].

Our study has several limitations. As in all surveys we relied on self-reported adherence to health behavior change, and it is possible that desirability bias may have led some respondents to over-estimate the degree to which they adhere. However, to mitigate this potential bias, we defined adherence as those who claimed that they made behavior changes “all of the time”, as has been done previously [35]. We also lacked some clinical data to fully identify those who might be considered at high cardiovascular risk and require annual monitoring tests, although our algorithm to define them was based on practice guidelines. For our disease monitoring variables, we were unable to distinguish between cases where physicians did not order recommended tests and cases where patients failed to follow through with receiving the tests. The sample size available for the reasons for non-adherence was a further limitation, with ‘personal choice’ being the only category we were able to consistently analyze for each outcome. Further, while our overall sample was sizeable (n = 1849), when stratified by income some outcomes were present in a limited number of respondents, yielding wide confidence intervals and low power to detect meaningful differences for some variables.

Despite these limitations, our study has important findings, and suggests potential areas for improvement with regard to the amount and quality of advice physicians give patients regarding health behavior change. Despite relatively frequent behavioral recommendations, non-adherence to this advice is generally high, suggesting that physician advice might be necessary but insufficient to motivate health behavior change.

Those with low income are at particularly high risk to fail to adhere to recommendations for smoking cessation and to receive monitoring tests. The detailed reasons for non-adherence or non-receipt of monitoring remain to be determined, although they were less likely to be related to personal choice, and more likely to be due to extrinsic factors. These observations suggest that potentially modifiable barriers may explain these different patterns of care in people of lower socioeconomic status.

## Supporting Information

Appendix S1 **Reasons for non-receipt of monitoring tests, or non-adherence to health behavior change.**
(DOCX)
