# Supplementary material for: The Association of Income with Health Behavior Change and Disease Monitoring among Patients with Chronic Disease
Source: PLoS One. 2014 Apr 10;9(4):e94007. doi: 10.1371/journal.pone.0094007 (PMC3983092; doi:10.1371/journal.pone.0094007)
Supplement: Appendix S1 — Reasons for non-receipt of monitoring tests, or non-adherence to health behavior change. (DOCX) [file pone.0094007.s001.docx]

| **Appendix S1 – Reasons for non-receipt of monitoring tests, or non-adherence to health behavior change** | | | | |
| --- | --- | --- | --- | --- |
| **Non-receipt of monitoring test** | | | **Non-adherence to health behavior change** | |
| **Type of Reason Provided** | | **Survey Responses** | **Type of Reason Provided** | **Survey Responses** |
| **Intrinsic** | **Personal Choice** | Have not gotten around to it  Did not think it was necessary  Personal/Family responsibilities | **Personal Choice** | Time constraints  Do not like to __  Lack of will power  Do not think it is important  No reason |
|  | **Patient Knowledge** | Did not know where to go  Fear | **Patient Knowledge** | Don’t know that it is safe or Recommended  Can’t control  Already taking medication for chronic disease |
| **Extrinsic** | **Financial/Cost** | Cost  Transportation | **Financial/Cost** | Too costly to eat low salt foods  Too costly to eat varied diet  Too costly to engage in physical activity  Cost of smoking cessation products  Too costly to control weight |
|  | **Not available/accessible** | Doctor did not think it was necessary  Doctor did not recommend it  Not available when required  Not available in the area  Waiting time was too long | **Not available/accessible** | Physical activity not available in area |
|  | **Other** | Unable to leave the house due to health problem  Language problem | **Other** | Physical condition or other health problem preventing activity  Tried to quit smoking, but failed  Feel that weight is normal (despite BMI>25) |
